# Long-Term Effects of Internet-Based Cognitive Behavioral Therapy on Depression Prevention Among University Students: Randomized Controlled Factorial Trial

**DOI:** 10.2196/56691

**Published:** 2024-09-24

**Authors:** Yukako Nakagami, Teruhisa Uwatoko, Tomonari Shimamoto, Masatsugu Sakata, Rie Toyomoto, Kazufumi Yoshida, Yan Luo, Nao Shiraishi, Aran Tajika, Ethan Sahker, Masaru Horikoshi, Hisashi Noma, Taku Iwami, Toshi A Furukawa

**Affiliations:** 1 Kyoto University Health Service, Kyoto University, Kyoto, Japan; 2 Department of Psychiatry, Graduate School of Medicine, Kyoto University, Kyoto, Japan; 3 University Health Center, Kyoto University of Education, Kyoto, Japan; 4 Department of Health Promotion and Human Behavior, Graduate School of Medicine / School of Public Health, Kyoto University, Kyoto, Japan; 5 Department of Neurodevelopmental Disorders, Graduate School of Medical Sciences, Nagoya City University, Nagoya, Japan; 6 Department of Psychiatry and Cognitive-Behavioral Medicine, Graduate School of Medical Science, Nagoya City University, Nagoya, Japan; 7 Population Health and Policy Research Unit, Medical Education Center, Graduate School of Medicine, Kyoto University, Kyoto, Japan; 8 National Centre for Cognitive Behavior Therapy and Research, National Centre of Neurology and Psychiatry, Tokyo, Japan; 9 Department of Interdisciplinary Statistical Mathematics, The Institute of Statistical Mathematics, Tokyo, Japan; 10 Office of Institutional Advancement and Communications, Kyoto University, Kyoto, Japan

**Keywords:** iCBT, depression prevention, student mental health, factorial randomized controlled trial, mobile phone

## Abstract

**Background:**

Internet-based cognitive behavioral therapy (iCBT) shows promise in the prevention of depression. However, the specific iCBT components that contribute to its effectiveness remain unclear.

**Objective:**

We aim to evaluate the effects of iCBT components in preventing depression among university students.

**Methods:**

Using a smartphone cognitive behavioral therapy (CBT) app, we randomly allocated university students to the presence or absence of 5 different iCBT components: self-monitoring, behavioral activation, cognitive restructuring, assertiveness training, and problem-solving. The active intervention lasted 8 weeks but the app remained accessible through the follow-up. The primary outcome was the onset of a major depressive episode (MDE) between baseline and the follow-up after 52 weeks, as assessed with the computerized World Health Organization Composite International Diagnostic Interview. Secondary outcomes included changes in the 9-item Patient Health Questionnaire, 7-item General Anxiety Disorder, and CBT Skills Scale.

**Results:**

During the 12-month follow-up, 133 of 1301 (10.22%) participants reported the onset of an MDE. There were no significant differences in the incidence of MDEs between the groups with or without each component (hazard ratios ranged from 0.85, 95% CI 0.60‐1.20, for assertiveness training to 1.26, 95% CI 0.88‐1.79, for self-monitoring). Furthermore, there were no significant differences in the changes on the 9-item Patient Health Questionnaire, 7-item General Anxiety Disorder, or for CBT Skills Scale between component allocation groups. However, significant reductions in depression and anxiety symptoms were observed among all participants at the 52-week follow-up.

**Conclusions:**

In this study, we could not identify any specific iCBT components that were effective in preventing depression or the acquisition of CBT skills over the 12-month follow-up period, but all participants with and without intervention of each iCBT component demonstrated significant improvements in depressive and anxiety symptoms. Further research is needed to explore the potential impact of frequency of psychological assessments, nonspecific intervention effects, natural change in the mental state, and the baseline depression level.

## Introduction

Major depressive disorder (MDD) is one of the most common mental disorders among university students with an estimated 12-month prevalence of 18.5% [1]. MDD can disrupt academic, interpersonal, and social functioning and can lead to suicide [2]. The suicide risk among people with MDD is more than 20 times greater than in the general population [2]. Worldwide, the World Health Organization (WHO) reports that suicide is the fourth leading cause of death among 15-29 year olds [3]. According to Ministry of Health, Labor and Welfare Suicide Countermeasures Promotion Office, in Japan, between 2020 and 2022, over 1000 students per year lost their lives to suicide, and suicide was the leading cause of death among those aged 15 to 34 years [4]. To improve functioning and to reduce suicide risk, preventing major depressive episodes (MDEs) among university students is especially important.

Several psychological interventions have shown promise for preventing depression [5] and cognitive behavioral therapy (CBT)–based strategies make up the bulk of the evidence [6,7]. Further, 1 recent systematic review showed that psychological interventions, mostly CBT-based, reduce the chances of depression incidence by 19% [8]. Furthermore, CBT-based depression prevention interventions are proven efficacious among child and adolescent populations [7]. Additionally, internet-based cognitive behavioral therapy (iCBT) has demonstrated consistent efficaciousness in treating depression [9,10]. iCBT is highly desirable because of its ease of accessibility and lower costs than face-to-face interventions [11].

However, the efficaciousness of iCBT has been examined as a package of several components [9]. In these packages, participants can receive specific modules from a menu of CBT skills, including psychoeducation, self-monitoring (SM), behavioral activation (BA), cognitive restructuring (CR), assertiveness training (AT), and problem-solving (PS). Hence, the efficacy of individual components for preventing depression remains unknown. By focusing on the effective components, iCBT could be conducted more efficiently, further boosting the scalability among university students.

In this large factorial trial, we aimed to identify the efficacious components of iCBT for preventing depression in the long-term. Our previous analysis of acute phase effects on depressive symptoms indicated an improvement in symptoms regardless of iCBT component allocation [12]. However, the long-term effects of each iCBT component on MDE incidence is still unclear. If reducing the occurrence of MDEs is due to specific iCBT components learned over the course of a year, this would indicate the necessary intervention ingredients needed to reduce long-term MDD progression.

## Methods

### Design

We conducted the Healthy Campus Trial, a fully factorial trial of 5 iCBT components: SM, BA, CR, AT, and PS. This study was designed as a fully factorial randomized controlled trial. The original protocol [13] explains the finer details of the Healthy Campus Trial.

### Participants

Undergraduate or graduate students from 5 universities in Japan were recruited between September 2018 and May 2021. Students aged between 18 and 39 years, possessing a smartphone, were introduced to online screening with the 9-item Patient Health Questionnaire (PHQ-9). To examine the effect of smartphone CBT on those with and without mild mental symptoms, based on the screening PHQ-9 scores, we included a random tenth of students scoring 4 or less on the PHQ-9 and all of the subthreshold depressive students with PHQ-9 scores between 5 and 9, or between 10 and 14 with suicidal ideation for less than half of the days. We were able to include only 1 in 10 of the students with no to very mild symptoms because including them all would have made the total sample size 10 times greater, and our budget and manpower could not afford this. Since the focus of the acute phase intervention [12,13] was on those with subthreshold depression, we prioritized their inclusion, while for the long-term outcome, we also wished to examine the effect of the smartphone CBT on those with or without mild symptoms. We excluded students under treatment by any mental health professionals. No student was diagnosed with an MDE at baseline.

### Interventions

All participants received a psychoeducation module. With psychoeducation, participants learned the importance of monitoring their own mental state through the trial duration using the smartphone app “Resilience Training,” which was developed for this study. After psychoeducation and baseline PHQ-9 assessment, iCBT combinations were randomly allocated for each participant among 32 combinations (with or without each of the 5 iCBT components, hence 2^5^=32 combinations, see Multimedia Appendix 1). Participants could be either assigned to or not assigned to each one individually. As a result, some participants might end up with zero iCBT components after the psychoeducation, while others could receive all 5. We used block randomization stratified by participant university and baseline PHQ-9 score (4 or less versus 5 or more). Each component was supposed to take 1 week. All participants, regardless of the number of the allocated components, were expected to finish the app in 8 weeks. Participants work on each component for 7‐10 days. They were able to review any assigned components as often as they liked during the 8 weeks and fill in as many additional worksheets as they wished. The participants had to complete (read all the lessons and complete at least one worksheet) each component before they could advance to the next component. Participants received digital gift cards worth US $9 for completing self-checks in weeks 4 and 24 and US $18 in weeks 8 and 52. In addition, research staff sent regular emails to encourage all participants to move forward with the program and reminder emails to fill in questionnaires if required. These emails did not include any specific guidance on the content of iCBT.

The contents of the five iCBT components were as follows:

1. SM: Participants monitored their own reactions to different situations (ie, feelings, thoughts, behaviors, and physical reactions) on mind maps and learned the association between each reaction.
2. BA: Participants recorded their activities with facilitation of new challenges through gamification in the “Action Marathon.”
3. CR: Participants recorded automatic thoughts and situations in a mind map. They were challenged to broaden their thoughts using several items, including telephone calls from imagined friends, a thought credibility percentage calculator, and a “Serenity Prayer”–modeled self-talk script.
4. AT: Participants learned assertive communication methods and recorded the content of real conversations, the situations, and the people involved.
5. PS: Participants set goals regarding difficult situations, raised ideas for solving associated problems, and evaluated the merits and demerits of each idea.

All CBT component lessons were delivered through the “Resilience Training” app.

### Measurements

The primary outcome was time to the first MDE by 52 weeks post randomization. The occurrence of an MDE was evaluated by the computerized version of the WHO Composite International Diagnostic Interview (CIDI) version 3.0 depression section, which has demonstrated good reliability [14,15]. The secondary outcomes included depressive symptoms, anxiety symptoms, and 5 CBT skills, as measured by the PHQ-9 [16,17], 7-item General Anxiety Disorder (GAD-7) [18,19], and CBT Skills Scale [20], respectively. The reliability of each questionnaire is well supported by previous evidence, including our study in which the CBT Skills Scale was developed and validated [20]. The timing for each questionnaire is detailed in the original protocol [13]. For the current analysis, the PHQ-9 was measured at baseline and at weeks 1 through 8, followed by every 4 weeks thereafter, up to week 52. The GAD-7 and the CBT Skills Scale were measured at baseline, and at the fourth, eighth, 24th, and 52nd weeks of this study. The CIDI results were obtained at week 52. To ensure participant safety, those with high levels of depression and suicidal ideation measured by the PHQ-9 were monitored and advised via email to seek professional care. All questionnaires were administered using the Japanese version.

### Statistical Analysis

We used SAS (version 9.4, SAS Institute) and R (version 4.2.1, R Foundation for Statistical Computing) for all analyses. We performed Cox regression analyses to investigate whether the presence or absence of each CBT component was related to the occurrence of an MDE. The model involves 5 iCBT components, baseline PHQ-9 scores, university, age, and sex as explanatory variables. Allocation of each iCBT component was defined based on an intention to treat (ITT). We plotted Kaplan-Meier survival curves for a graphical representation of MDE-free survival for each iCBT component. Interval censorings were addressed with Anderson-Bergman adjustment methods [21,22]. In addition, changes in PHQ-9, GAD-7, and CBT Skills Scale were repeatedly measured up to 52 weeks and analyzed with mixed models for repeated measures [23]. The correlation matrix of repeated outcomes was assumed to be unstructured. For explanatory variables, 5 iCBT components, week, iCBT component by week interaction, university, age, and baseline scores were modeled. The between-group effect size was estimated by dividing the estimated mean difference by the observed SD of week 52 scores, which is the most widely used definition of standardized difference in clinical trials of psychiatry [24]. In addition, the within-group effect size was estimated by dividing the estimated mean change by the baseline SD.

### Ethical Considerations

This study involves human participants and has been approved by the Ethics Committee of the Kyoto University School of Medicine (C1357). All data obtained from participants were deidentified, and participants provided informed consent before participating in this study.

## Results

### Demographics

**Figure 1.**
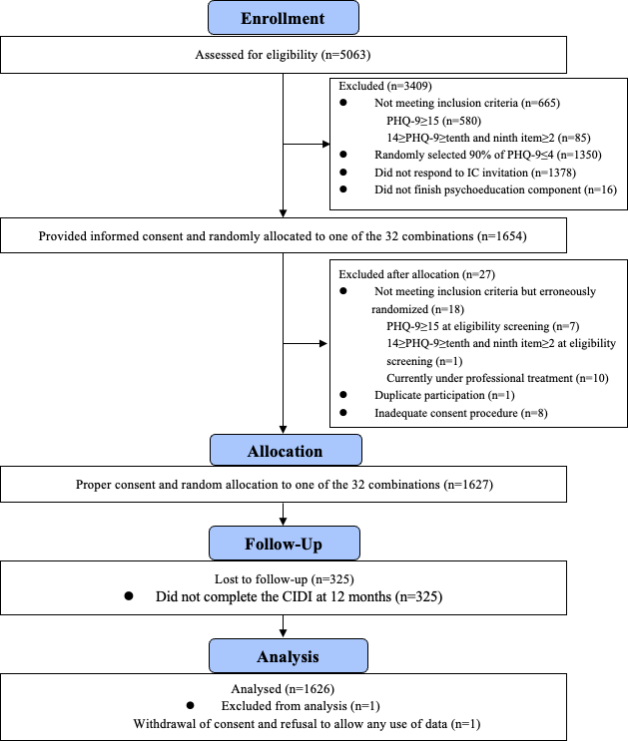
CONSORT diagram. CIDI: Composite International Diagnostic Interview; CONSORT: Consolidated Standards of Reporting Trials; IC: informed consent; PHQ-9: 9-item Patient Health Questionnaire.

Figure 1 presents the CONSORT diagram. Of the 5063 students assessed for eligibility, 1627 students provided informed consent and were randomly allocated to 1 of the 32 iCBT combinations (Multimedia Appendix 1). Finally, 1626 students were included in this study, after 1 student withdrew consent and refused the use of their data for analysis. Table 1 tabulates the demographic characteristics of the participants. The mean age was 21.5 (SD 3.0) years, and 933 (57.38%) were women. The mean PHQ-9 and GAD-7 scores were 6.4 (SD 3.4) and 5.5 (SD 3.4), respectively. Participants were allocated to each module (SM: n=808; BA: n=817; CR: n=811; AT: n=814; PS: n=811). The characteristics of the participants per component are described in Multimedia Appendix 2, and the number of participants allocated to each of the 32 combinations is described in Multimedia Appendix 1.

**Table 1.** Baseline characteristics of total participants for 1-year analysis (N=1626).

				Participants
**Demographics**				
Age (years), mean (SD)				21.5 (2.99)
Sex (female), n (%)				933 (57.38)
Undergraduate, n (%)				1250 (76.88)
Married, n (%)				33 (2.03)
Living alone (apart from family), n (%)				1002 (61.62)
Part-time employment, n (%)				1261 (77.55)
Smoking experience, n (%)				94 (5.78)
Drinking alcohol regularly, n (%)				665 (40.90)
Exercise opportunity, n (%)				1014 (62.36)
History of psychiatric or psychological treatment, n (%)				184 (11.32)
History of major depressive episode in past year (CIDI^a^), n (%)				151 (9.29)
**Cognitive and behavioral skills, mean (SD)**				
SM^b^ skills				8.03 (3.26)
BA^c^ skills				10.53 (4.33)
CR^d^ skills				8.71 (3.44)
AT^e^ skills				9.60 (3.55)
PS^f^ skills				10.6 (3.09)
**Clinical characteristic, mean (SD)**				
PHQ-9^g^				6.38 (3.40)
GAD-7^h^				5.47 (3.35)
**Function, mean (SD)**				
WHO-HPQ^i^ Presenteeism				6.30 (1.58)

a CIDI: Composite International Diagnostic Interview.

b SM: self-monitoring.

c BA: behavioral activation.

d CR: cognitive restructuring.

e AT: assertiveness training.

f PS: problem-solving.

g PHQ-9: 9-item Patient Health Questionnaire.

h GAD-7: 7-item General Anxiety Disorder.

i WHO-HPQ: World Health Organization Health and Work Performance Questionnaire.

### Primary Analysis

**Figure 2.**
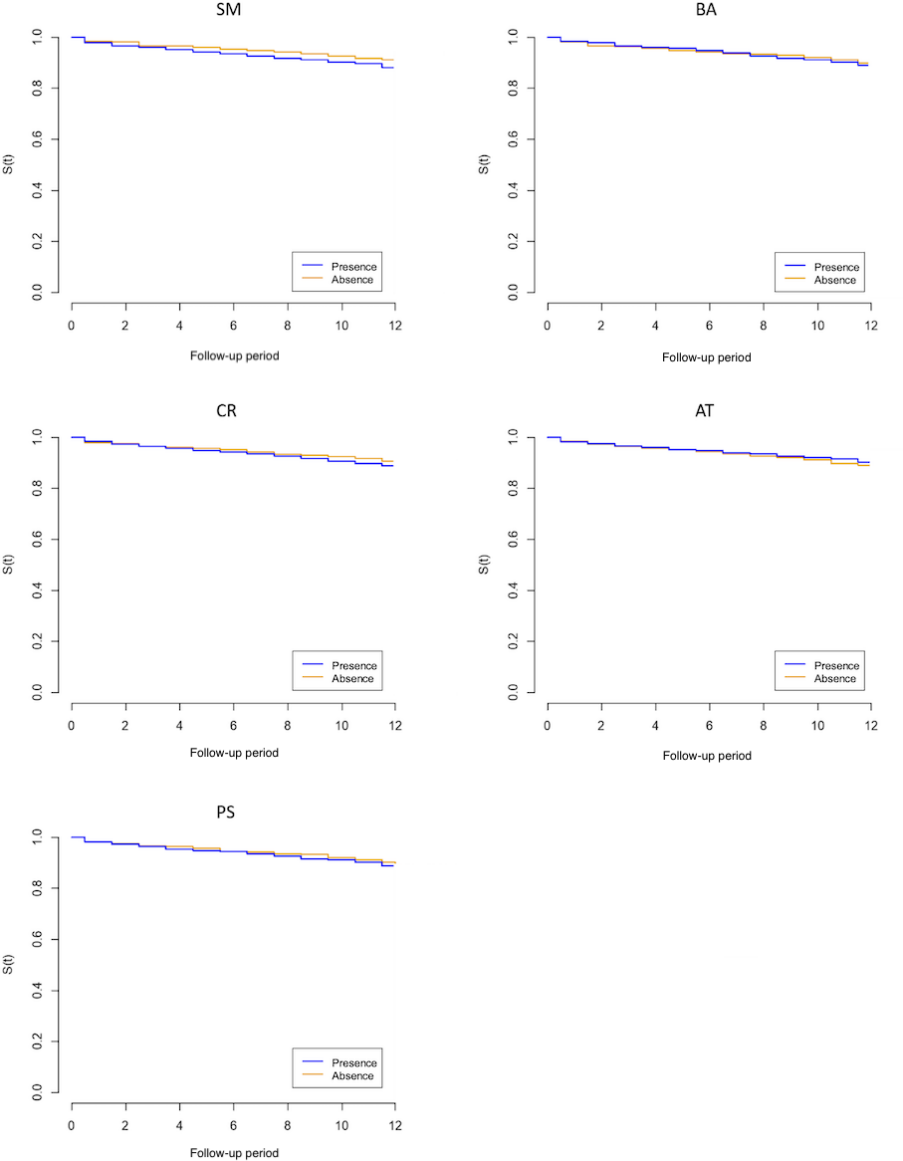
Survival curves for MDEs for the 5 CBT components. AT: assertiveness training; BA: behavioral activation; CBT: cognitive behavioral therapy; CR: cognitive restructuring; MDE: major depressive episode; PS: problem-solving; S(t): survival function (major depressive episode–free survival); SM: self-monitoring.

Of the 1626 study participants, 1301 (80.01%) responded to the 52-week follow-up survey. Among the responders, 133 (10.22%) reported incident MDE by week 52. The number of completions and their percentages for each assigned module were recorded (SM: 743/808, 92.6%; BA: 703/817, 86%; CR: 688/811, 84.8%; AT: 680/814, 83.8%; PS: 666/811, 82.1%). Table 2 summarizes the results of the multivariable Cox regression analysis of each CBT component on MDE occurrence. The hazard ratios (95% CI) of presence over absence were 1.26 (0.88‐1.79) for SM, 1.09 (0.77‐1.54) for BA, 1.18 (0.83‐1.68) for CR, 0.85 (0.60‐1.20) for AT, and 1.18 (0.82‐1.68) for PS. Multimedia Appendix 3 shows the full results of the analysis with coefficients for the included covariates. The results of the univariable Cox regression analysis (Multimedia Appendix 4) showed similar results to those of the multivariable analyses. Figure 2 presents the Kaplan-Meier survival curves for the occurrence of an MDE for each of the 5 iCBT components. We did not find a significant difference in the occurrence of an MDE in each iCBT component.

**Table 2.** Summary of the results of multiple cox regression analyses of each CBT^a^ component on occurrence of an MDE^b^ the results of the analysis for each component are shown in Multimedia Appendix 3.^c^

Component	Cases with MDE, n/N (completed cases)		HR^d^ for incident MDE					
	Presence	Absence	HR	SE	95% CI			*P* value
SM^e^	75/808 (638)	58/818 (663)	1.26	0.18	0.88-1.79			.21
BA^f^	69/817 (648)	64/809 (653)	1.09	0.18	0.77-1.54			.63
CR^g^	72/811 (652)	61/815 (649)	1.18	0.18	0.83-1.68			.37
AT^h^	62/814 (647)	71/812 (654)	0.85	0.18	0.60-1.20			.35
PS^i^	71/811 (646)	62/815 (655)	1.18	0.18	0.82-1.68			.37

a CBT: cognitive behavioral therapy.

b MDE: major depressive episode

c Age, sex, baseline 9-item Patient Health Questionnaire point, and recruition site (4 universities) were adjusted in the Cox regression analysis.

d HR: hazard ratio.

e SM: self-monitoring.

f BA: behavioral activation.

g CR: cognitive restructuring.

h AT: assertiveness training.

i PS: problem-solving.

### Secondary Analysis

Table 3 shows the estimated least squares mean change scores of the PHQ-9 for participants allocated to the presence or absence of each component using the mixed models for repeated measures. Compared to the baseline, the estimated least squares mean change scores ranged between −1.77 and −1.97 at the 52-week follow-up for both the presence and absence groups across all iCBT components. Regardless of the allocated iCBT component, average depressive symptoms were reduced from baseline. However, no significant differences in change scores were found in the comparison between the presence group and the absence group across all iCBT components. Analysis of the GAD-7 showed similar results for anxiety symptoms, as shown in Table 4. Table 5 shows an analysis of the relevant CBT skills at week 52 for each CBT component. There was no difference in changes in CBT skills between the presence or absence of each iCBT component.

**Table 3.** Summary of the results of differences of change scores of PHQ-9^a^ at 52 weeks using MMRM^b^ CBT^c^ components, week, CBT component by week interaction, university, age, and baseline scores as fixed effects, while participants were introduced as a random effect. Effect size was estimated by division of the estimated mean changes by the observed SD of baseline scores.

Component		Participants, n	Estimated least squares mean change scores of PHQ-9 within groups (95% CI)	Within-group effect size for baseline-week 52 change scores of PHQ-9 (95% CI)	Estimated differences of baseline-week 52 change scores between groups (95% CI)	Effect size for differences of baseline-week 52 change scores between groups (95% CI)	*P* values
**SM** ^**d**^					−0.09 (−0.28 to 0.4)	−0.02 (−0.06 to 0.09)	.81
	Presence	808	−1.91 (−2.43 to −1.39)	−0.69 (−0.87 to −0.50)			
	Absence	818	−1.83 (−2.35 to −1.31)	−0.66 (−0.84 to −0.47)			
**BA** ^**e**^					−0.14 (−0.43 to 0.25)	−0.03 (−0.09 to 0.05)	.71
	Presence	817	−1.94 (−2.46 to −1.41)	−0.70 (−0.88 to −0.51)			
	Absence	809	−1.8 (−2.32 to −1.28)	−0.65 (−0.83 to −0.46)			
**CR** ^**f**^					0.21 (−0.29 to 0.39)	0.04 (−0.06 to 0.08)	.58
	Presence	811	−1.77 (−2.28 to −1.25)	−0.64 (−0.82 to −0.45)			
	Absence	815	−1.97 (−2.50 to −1.45)	−0.71 (−0.90 to −0.52)			
**AT** ^**g**^					0.21 (−0.16 to 0.52)	0.04 (−0.04 to 0.11)	.57
	Presence	814	−1.77 (−2.29 to −1.24)	−0.64 (−0.82 to −0.45)			
	Absence	812	−1.97 (−2.49 to −1.45)	−0.71 (−0.90 to −0.52)			
**PS** ^**h**^					−0.09 (−0.30 to 0.38)	−0.02 (−0.06 to 0.08)	.80
	Presence	811	−1.92 (−2.43 to −1.40)	−0.69 (−0.87 to −0.50)			
	Absence	815	−1.82 (−2.35 to −1.3)	−0.65 (−0.84 to −0.47)			

a PHQ-9: 9-item Patient Health Questionnaire.

b MMRM: mixed models for repeated measures.

c CBT: cognitive behavioral therapy.

d SM: self-monitoring.

e BA: behavioral activation.

f CR: cognitive restructuring.

g AT: assertiveness training.

h PS: problem-solving.

**Table 4.** Summary of the results of differences of change scores of GAD-7^a^ at 52 weeks using MMRM^b^ CBT^c^ components, week, CBT component by week interaction, university, age, and baseline scores as fixed effects, while participants were introduced as a random effect. Effect size was estimated by division of the estimated mean changes by the observed SD of baseline scores.

Component		Participants, n	Estimated least squares mean change scores of GAD-7 within groups (95% CI)	Within-group effect size for baseline-week 52 change score of GAD-7 (95% CI)	Estimated differences of baseline-week 52 change scores between groups (95% CI)	Effect size for differences of baseline-week 52 change scores between groups (95% CI)	*P* values
**SM** ^**d**^					0.27 (−0.46 to 1)	0.08 (−0.14 to 0.3)	.47
	Presence	808	−1.28 (−1.82 to −0.74)	−0.38 (−0.55 to −0.22)			
	Absence	818	−1.55 (−2.08 to −1.02)	−0.47 (−0.62 to −0.31)			
**BA** ^**e**^					−0.32 (−1.04 to 0.41)	−0.09 (−0.31 to 0.12)	.39
	Presence	817	−1.57 (−2.11 to −1.04)	−0.47 (−0.63 to −0.31)			
	Absence	809	−1.26 (−1.79 to −0.72)	−0.38 (−0.54 to −0.22)			
**CR** ^**f**^					0.30 (−0.43 to 1.02)	0.09 (−0.13 to 0.31)	.42
	Presence	811	−1.27 (−1.79 to −0.74)	−0.38 (−0.54 to −0.22)			
	Absence	815	−1.56 (−2.11 to −1.02)	−0.47 (−0.63 to −0.31)			
**AT** ^**g**^					0.14 (−0.59 to 0.86)	0.04 (−0.18 to 0.26)	.71
	Presence	814	−1.35 (−1.89 to −0.81)	−0.41 (−0.57 to −0.24)			
	Absence	812	−1.48 (−2.01 to −0.95)	−0.44 (−0.60 to −0.29)			
**PS** ^**h**^					−0.01 (−0.73 to 0.72)	0 (−0.22 to 0.22)	.99
	Presence	811	−1.42 (−1.95 to −0.88)	−0.43 (−0.59 to −0.26)			
	Absence	815	−1.41 (−1.95 to −0.87)	−0.42 (−0.59 to −0.26)			

a GAD-7: 7-item General Anxiety Disorder.

b MMRM: mixed models for repeated measures.

c CBT: cognitive behavioral therapy.

d SM: self-monitoring.

e BA: behavioral activation.

f CR: cognitive restructuring.

g AT: assertiveness training.

h PS: problem-solving.

**Table 5.** Summary of the estimated least squares mean differences of change in corresponding CBT^a^ skills at 52 weeks using MMRM^b^ in addition to CBT component, week, CBT component by week interaction, university, age, and baseline scores as fixed effects, while participants were introduced as a random effect.

Component	Estimated least squares mean differences of change in corresponding CBT skill (95% CI)
SM^c^	0.23 (−0.28 to 0.75)
BA^d^	−0.44 (−1.10 to 0.22)
CR^e^	−0.1 (−0.58 to 0.38)
AT^f^	0.14 (−0.39 to 0.66)
PS^g^	0.24 (−0.15 to 0.64)

a CBT: cognitive behavioral therapy.

b MMRM: mixed models for repeated measures.

c SM: self-monitoring.

d BA: behavioral activation.

e CR: cognitive restructuring.

f AT: assertiveness training.

g PS: problem-solving.

## Discussion

### Principal Findings

We conducted a fully factorial randomized trial of 5 iCBT skills to reduce the incidence of MDEs among university students. We did not observe significant preventive effects attributable to individual iCBT components in depression prevention. Secondary analyses showed no significant difference in change scores for PHQ-9, GAD-7, and CBT skills, regardless of the presence or absence of each iCBT component either. Participants exhibited improvements in depression and anxiety symptoms from the baseline to the 52 weeks, irrespective of the specific iCBT component allocation.

The significantly better PHQ-9 and GAD-7 scores compared to baseline at 52 weeks, irrespective of the iCBT component, replicate the results from our previous 8-week short-term analysis [12]. Multiple extra-intervention factors may have contributed to the general improvement effects. First, the frequent psychological assessments administered to all participants may have introduced a significant nonspecific therapeutic effect. This hypothesis is supported by findings from a prior meta-analysis of antidepressant efficacy trials, which demonstrated that follow-up assessments significantly influence placebo responses [25]. In this study, frequent follow-up visits significantly impacted depressive scale scores, where the extra follow-up visits accounted for approximately 34%‐44% of the overall placebo response observed over the course of the trials. These results emphasize that the therapeutic effect of these assessments is not only statistically significant but also cumulative and proportional to the number of interactions, thereby suggesting a potent nonspecific therapeutic effect stemming from the assessment frequency itself [25].

In our study, a rigorous schedule of assessments was implemented, occurring as many as 19 times throughout the duration of this study (specifically at weeks 1-8, 12, 16, 20, 24, 28, 32, 36, 40, 44, 48, and 52). This high frequency of assessments might have played a significant role in mediating a therapeutic effect, potentially confounding the distinct impacts attributable to each individual iCBT component. It is important to note, however, that all participants in this study were subjected to the same number of assessments, making it challenging to isolate the effects of the assessments from those of the iCBT interventions themselves. Consequently, while it is tempting to speculate about the influence of frequent assessments, such speculation cannot be conclusively confirmed given the uniform assessment protocol applied across all participants.

Given these considerations, it is recommended that future research should explore the implications of varying the frequency of assessments by including experimental groups subjected to differing assessment schedules. This approach would allow for a more refined analysis of how frequent interactions might influence outcomes, thereby enabling a more accurate assessment of the true efficacy of the iCBT components. By distinguishing the effects of the intervention from those of the assessment frequency, future studies could yield more reliable and generalizable findings.

In addition to frequent assessments, application help notifications coupled with encouragement emails may have produced extra-intervention improvement effects. Regardless of allocated iCBT components, participants were instructed to keep the app installed until the end of the 52-week follow-up. This instruction also conveyed crisis management information, such as where to go for help in case of serious problems. Providing emergency contact information was reported to reduce rates of repeated self-harm [26]. Thus, it is possible that the crisis management information in our study could have contributed positively to mental health conditions. Future studies should consider incorporating groups with different frequencies of self-assessments to derive more reliable results.

Together with the frequent follow-up assessments and application help notifications, university grade level could have affected the results. A longitudinal study reported that depressive and anxiety symptoms were better in the fourth year of university than in the first 3 years [27]. This trend toward improvement may mask the effect of our iCBT intervention. Similarly, baseline depression level may have affected our results. As depression symptoms become milder, placebo effects become more prominent relative to the intervention. This assertion is supported by a meta-analysis examining the effects of antidepressants compared to placebo [28]. If we targeted university students with more severe depression symptoms, we may have a greater ability to detect intervention effects. On the other hand, another study assessing the effects of an iCBT program with healthy participants showed a significant improvement in subthreshold depressive symptoms [10]. Therefore, our failure to identify the effects of the intervention may not be attributed solely to the depressive severity of the participants.

The latter half of our study unfolded during the COVID-19 pandemic, accompanied by extensive social restrictions. This period has been scrutinized across various studies, notably in a systematic review of epidemiological research which reported a temporary surge in depression and anxiety within the first 3 months of the pandemic [29]. However, our own secondary analysis, using the same data set [30], did not find a statistically significant link between depressive symptoms, as measured by the PHQ-9, and the implementation of 4 distinct states of emergency in Japan aimed at mitigating the spread of the virus. Although our results suggest no direct correlation between these emergency measures and depressive symptoms, the overarching impact of the pandemic—its indirect influence on mental health through other unmeasured factors—remains a plausible explanation for variations in our findings. Consequently, we acknowledge the complexity of the pandemic’s effects, which may interact with psychological outcomes in nuanced and varied ways that are not immediately apparent in our data.

The validated CBT Skills Scale [20] was used to measure the main 5 CBT skills. The results at 52 weeks showed no significant difference in change scores with or without each of the iCBT components. This finding was unexpected as our previously reported short-term analysis revealed significant improvement of CR and AT skills [12]. The 52-week results lack of significant differences may be due to the attenuation of skills learned during the first 8-week intervention period. However, some studies reported that the effect of CBT lasts longer than 46 months as an adjunct to pharmacotherapy for treatment-resistant depression [31]. These studies focused only on symptoms, not on CBT skills. Our report is the first to assess the persistence of CBT skills using our original CBT Skills Scale. Further research is needed to determine how long CBT skills are usually maintained. It is possible that maintenance CBT may be effective in maintaining CBT skills, despite the reported fact that maintenance CBT on depression prevention is superior to psychoeducation only among patients with a high risk of depression relapse [32].

### Limitations

There are several limitations in our study. First, our participant pool was limited to 2 prefectures in Japan (ie, Kyoto and Aichi). Nonetheless, this well-powered multisite trial experienced minimal attrition and included 3 types of universities, national, public, and private, which attracted students from all regions of Japan. Additionally, we ensured adequate allocation concealment during randomization, resulting in a well-distributed sample. Nevertheless, caution is advised when generalizing these results. Second, our study did not account for potentially significant protective factors, such as good academic performance and financial stability. Although our research used a fully factorial design intended to evenly distribute unknown confounders across study groups, we cannot definitively exclude the influence of unmeasured variables. Third, our measures of MDEs, mental state, and CBT skills relied on self-administered questionnaires that, despite their established reliability, are susceptible to biases such as social-desirability. This susceptibility potentially obscures the true symptoms of depression, and the accuracy of the PHQ-9 may also be compromised by the repeated measurement effect. To mitigate these limitations and enhance assessment accuracy, face-to-face evaluations conducted by trained professionals would be more ideal. Finally, the validated CBT Skills Scale used in our study may not have been sensitive enough to capture subtle differences in skill acquisition and retention over the extensive follow-up period. While the scale is robust for assessing general CBT competency, it may not adequately reflect incremental changes or the long-term retention of specific skills. Future research should use such tools to ensure a more precise and sensitive evaluation of the long-term skill acquisition and retention of CBT skills.

### Conclusions

This study did not show the specific impacts of individual components of iCBT on preventing MDEs. However, participants’ scores on the PHQ-9 and GAD-7 generally improved over the year during their involvement in this study. In addition to the statistical phenomenon of regression to the mean and the natural course of the conditions, the psychoeducation given to all participants, the frequent psychological assessments, and the experience of being involved in this study itself may have contributed to some alleviation of depression and anxiety symptoms among university students. Future iCBT optimization trials should consider psychological assessment frequency, the impact of common intervention elements, the natural history of the mental state of the target group, and the baseline depression level.

## Supplementary material

10.2196/56691 Multimedia Appendix 1 Combinations of cognitive and behavioral therapy skill components of cognitive behavioral therapy smartphone app.

10.2196/56691 Multimedia Appendix 2 Baseline characteristics of total participants for 1-year analysis (N=1626) and by each component.

10.2196/56691 Multimedia Appendix 3 Results of multiple Cox regression analyses.

10.2196/56691 Multimedia Appendix 4 Results of simple Cox regression analyses.

10.2196/56691 Checklist 1 CONSORT-eHEALTH checklist (V 1.6.1).

## Abbreviations

AT: assertiveness training
BA: behavioral activation
CBT: cognitive behavioral therapy
CR: cognitive restructuring
GAD-7: 7-item General Anxiety Disorder
iCBT: internet-based cognitive behavioral therapy
ITT: intention-to-treat
MDD: major depressive disorder
MDE: major depressive episode
PHQ-9: 9-item Patient Health Questionnaire
PS: problem-solving
SM: self-monitoring
WHO: World Health Organization
