# Supplementary material for: Long-Term Effects of Internet-Based Cognitive Behavioral Therapy on Depression Prevention Among University Students: Randomized Controlled Factorial Trial
Source: JMIR Ment Health. 2024 Sep 24;11:e56691. doi: 10.2196/56691 (PMC11445681; doi:10.2196/56691)
Supplement: Multimedia Appendix 1 [file mental-v11-e56691-s001.pdf]

	PE	SM	CR	BA	AT	PS	Order								n
C1	1	1	1	1	1	1	PE	AT	PS	SM	CR	BA			27
C2	1	1	1	1	1	1	PE			SM	BA	CR	AT	PS	25
C3	1	1	1	1	1	0	PE			SM	CR	BA	AT		26
C4	1	1	1	1	1	0	PE	AT		SM	BA	CR			26
C5	1	1	1	1	0	1	PE		PS	SM	CR	BA			25
C6	1	1	1	1	0	1	PE			SM	BA	CR		PS	25
C7	1	1	1	1	0	0	PE			SM	CR	BA			26
C8	1	1	1	1	0	0	PE			SM	BA	CR			25
C9	1	1	1	0	1	1	PE			SM	CR		AT	PS	26
C10	1	1	1	0	1	1	PE	AT	PS	SM	CR				23
C11	1	1	1	0	1	0	PE			SM	CR		AT		25
C12	1	1	1	0	1	0	PE	AT		SM	CR				24
C13	1	1	1	0	0	1	PE			SM	CR			PS	24
C14	1	1	1	0	0	1	PE		PS	SM	CR				25
C15	1	1	1	0	0	0	PE			SM	CR				25
C16	1	1	1	0	0	0	PE			SM	CR				26
C17	1	1	0	1	1	1	PE			SM		BA	AT	PS	26
C18	1	1	0	1	1	1	PE	AT	PS	SM		BA			25
C19	1	1	0	1	1	0	PE			SM		BA	AT		25
C20	1	1	0	1	1	0	PE	AT		SM		BA			26
C21	1	1	0	1	0	1	PE			SM		BA		PS	23
C22	1	1	0	1	0	1	PE		PS	SM		BA			26
C23	1	1	0	1	0	0	PE			SM		BA			24
C24	1	1	0	1	0	0	PE			SM		BA			26
C25	1	1	0	0	1	1	PE			SM			AT	PS	24
C26	1	1	0	0	1	1	PE	AT	PS	SM					26
C27	1	1	0	0	1	0	PE			SM			AT		27
C28	1	1	0	0	1	0	PE	AT		SM					25
C29	1	1	0	0	0	1	PE			SM				PS	25
C30	1	1	0	0	0	1	PE		PS	SM					26
C31	1	1	0	0	0	0	PE			SM					23
C32	1	1	0	0	0	0	PE			SM					28
C33	1	0	1	1	1	1	PE				CR	BA	AT	PS	25
C34	1	0	1	1	1	1	PE	AT	PS		BA	CR			25
C35	1	0	1	1	1	0	PE	AT			CR	BA			27
C36	1	0	1	1	1	0	PE				BA	CR	AT		24
C37	1	0	1	1	0	1	PE				CR	BA		PS	27
C38	1	0	1	1	0	1	PE		PS		BA	CR			25
C39	1	0	1	1	0	0	PE				CR	BA			26
C40	1	0	1	1	0	0	PE				BA	CR			24
C41	1	0	1	0	1	1	PE				CR		AT	PS	26
C42	1	0	1	0	1	1	PE	AT	PS		CR				26
C43	1	0	1	0	1	0	PE				CR		AT		26
C44	1	0	1	0	1	0	PE	AT			CR				23
C45	1	0	1	0	0	1	PE				CR			PS	29
C46	1	0	1	0	0	1	PE		PS		CR				26
C47	1	0	1	0	0	0	PE				CR				26
C48	1	0	1	0	0	0	PE				CR				23
C49	1	0	0	1	1	1	PE					BA	AT	PS	26
C50	1	0	0	1	1	1	PE	AT	PS			BA			24
C51	1	0	0	1	1	0	PE					BA	AT		29
C52	1	0	0	1	1	0	PE	AT				BA			24
C53	1	0	0	1	0	1	PE					BA		PS	26
C54	1	0	0	1	0	1	PE		PS			BA			26
C55	1	0	0	1	0	0	PE					BA			28
C56	1	0	0	1	0	0	PE					BA			25
C57	1	0	0	0	1	1	PE						AT	PS	26
C58		0	0	0	1	1	PE	AT	PS						23
C59	1	0	0	0	1	0	PE						AT		26
C60	1	0	0	0	1	0	PE	AT							28
C61	1	0	0	0	0	1	PE							PS	25
C62	1	0	0	0	0	1	PE		PS						24
C63	1	0	0	0	0	0	PE								26
C64	1	0	0	0	0	0	PE								24
															1626

Note: For the overall Healthy Campus Trial, 64 groups were constructed by combining five components and one type of reordering to see the order effect of BA and CR. In the present study, the analysis was conducted as 32 groups to examine the effects of the five components only. AT: assertiveness training, BA: behavioral activation, CR: cognitive restructuring, PS: problem solving, SM: self-monitoring; half-tone dot meshing cells denote the combination where the ordering of CR and BA is reversed
