# Supplementary material for: Long-Term Effects of Internet-Based Cognitive Behavioral Therapy on Depression Prevention Among University Students: Randomized Controlled Factorial Trial
Source: JMIR Ment Health. 2024 Sep 24;11:e56691. doi: 10.2196/56691 (PMC11445681; doi:10.2196/56691)
Supplement: Multimedia Appendix 2 [file mental-v11-e56691-s002.pdf]

Baseline characteristics of total participants for 1-year analysis (N=1626) and by each component

		SM[a]		BA[b]		CR[c]		AT[d]		PS[e]	
	Total	Presence	Absence	Presence	Absence	Presence	Absence	Presence	Absence	Presence	Absence
N	1626	808	818	817	809	811	815	814	812	811	815
Demographic											
Age (years), mean (SD)	21.50(2.99)	21.56(3.01)	21.48(2.96)	21.54(2.86)	21.50(3.11)	21.56(3.09)	21.48(2.87)	21.52(3.12)	21.52(2.85)	21.53(3.08)	21.51(2.88)
Sex (female), n (%)	933(57.38)	463(57.3)	470(57.5)	464(56.8)	469(58.0)	462(57.0)	471(57.8)	470(57.7)	463(57.0)	476(58.7)	457(56.1)
Undergraduate, n (%)	1250(76.88)	613(75.9)	637(77.9)	626(76.6)	624(77.1)	630(77.7)	620(76.1)	627(77.0)	623(76.7)	619(76.3)	631(77.4)
Married, n (%)	33(2.03)	16(2.0)	17(2.1)	11(1.3)	22(2.7)	15(1.8)	18(2.2)	17(2.1)	16(2.0)	14(1.7)	19(2.3)
Living alone (apart from family), n (%)	1002(61.62)	510(63.1)	492(60.1)	514(62.9)	488(60.3)	500(61.7)	502(61.6)	505(62.0)	497(61.2)	504(62.1)	498(61.1)
Part-time employment, n (%)	1261(77.55)	615(76.1)	646(79.0)	646(79.1)	615(76.0)	624(76.9)	637(78.2)	623(76.5)	638(78.6)	635(78.3)	626(76.8)
Smoking experience, n (%)	94(5.78)	49(6.1)	45(5.5)	44(5.4)	50(6.2)	46(5.7)	48(5.9)	40(4.9)	54(6.7)	51(6.3)	43(5.3)
Drinking alcohol regularly, n (%)	665(40.90)	329(40.7)	336(41.1)	340(41.6)	325(40.2)	334(41.2)	331(40.6)	337(41.4)	328(40.4)	328(40.4)	337(41.3)
Exercise opportunity, n (%)	1014(62.36)	503(62.3)	511(62.5)	513(62.8)	501(61.9)	503(62.0)	511(62.7)	499(61.3)	515(63.4)	518(63.9)	496(60.9)
History of psychiatric / psychological treatment, n (%)	184(11.32)	102(12.6)	82(10.0)	92(11.3)	92(11.4)	85(10.5)	99(12.1)	88(10.8)	96(11.8)	84(10.4)	100(12.3)
History of major depressive episode in past year (CIDI[f]), n (%)	151(9.29)	84(10.4)	67(8.2)	74(9.1)	77(9.5)	79(9.7)	72(8.8)	68(8.4)	83(10.2)	76(9.4)	75(9.2)

Baseline characteristics of total participants for 1-year analysis (N=1626) and by each component

		SM[a]		BA[b]		CR[c]		AT[d]		PS[e]	
	Total	Presence	Absence	Presence	Absence	Presence	Absence	Presence	Absence	Presence	Absence
N	1626	808	818	817	809	811	815	814	812	811	815
Cognitive and behavioral skills											
SM Skills	8.03(3.26)	7.91(3.34)	8.15(3.15)	8.00(3.23)	8.06(3.28)	7.99(3.28)	8.07(3.22)	8.03(3.29)	8.03(3.21)	8.02(3.20)	8.04(3.30)
BA Skills	10.53(4.33)	10.58(4.46)	10.48(4.18)	10.45(4.37)	10.61(4.27)	10.66(4.28)	10.4(4.36)	10.56(4.30)	10.5(4.35)	10.57(4.25)	10.49(4.39)
CR Skills	8.71(3.44)	8.69(3.47)	8.72(3.41)	8.79(3.39)	8.61(3.49)	8.84(3.47)	8.57(3.40)	8.88(3.42)	8.52(3.44)	8.61(3.56)	8.8(3.31)
AT Skills	9.60(3.55)	9.44(3.56)	9.75(3.52)	9.61(3.44)	9.58(3.64)	9.60(3.59)	9.59(3.50)	9.64(3.48)	9.56(3.60)	9.44(3.59)	9.75(3.49)
PS Skills	10.60(3.09)	10.46(3.13)	10.73(3.03)	10.55(3.04)	10.65(3.12)	10.65(3.07)	10.55(3.09)	10.77(3.09)	10.43(3.07)	10.47(3.18)	10.72(2.97)
Clinical characteristic, mean (SD)											
PHQ-9[g]	6.38(3.40)	6.48(3.47)	6.28(3.32)	6.43(3.44)	6.32(3.35)	6.36(3.32)	6.39(3.48)	6.40(3.45)	6.35(3.34)	6.36(3.33)	6.39(3.47)
GAD-7[h]	5.47(3.35)	5.39(3.19)	5.55(3.49)	5.73(3.36)	5.20(3.30)	5.48(3.34)	5.46(3.35)	5.55(3.40)	5.39(3.28)	5.53(3.24)	5.40(3.44)
Function, mean (SD)											
WHO-HPQ[i] Presenteeism	6.3(1.58)	6.28(1.51)	6.32(1.64)	6.26(1.57)	6.34(1.58)	6.27(1.60)	6.33(1.55)	6.32(1.59)	6.28(1.56)	6.27(1.6)	6.32(1.54)

[a]SM: self-monitoring.

[b]BA: behavioral activation.

[c]CR: cognitive restructuring.

[d]AT: assertiveness training.

[e]PS: problem-solving.

[f]CIDI: Composite International Diagnostic Interview.

[g]PHQ-9: Patient Health Questionnaire-9.

[h]GAD-7: General Anxiety Disorder-7.

[i]WHO-HPQ: World Health Organization Health and Work Performance Questionnaire.
