# Supplementary material for: Long-Term Effects of Internet-Based Cognitive Behavioral Therapy on Depression Prevention Among University Students: Randomized Controlled Factorial Trial
Source: JMIR Ment Health. 2024 Sep 24;11:e56691. doi: 10.2196/56691 (PMC11445681; doi:10.2196/56691)
Supplement: Multimedia Appendix 3 [file mental-v11-e56691-s003.pdf]

Results of multiple Cox regression analyses of each CBT[a] component and covariates on the occurrence of MDE[b]

	HR[c]	SE[d]	95%CI			*P* value
SM[e]	1.26	0.18	0.88	to	1.80	.21
PHQ-9[f] Point	1.18	0.03	1.11	to	1.24	.00
university A	1.14	0.24	0.71	to	1.84	.58
university B	1.15	0.38	0.55	to	2.40	.71
university C	1.07	0.31	0.59	to	1.96	.82
university D	0.66	0.59	0.21	to	2.08	.48
age	1.03	0.03	0.97	to	1.09	.30
sex (male vs. female)	0.96	0.18	0.68	to	1.36	.82

	HR	SE	95%CI			*P* value
BA[g]	1.09	0.18	0.77	to	1.54	.63
PHQ-9 Point	1.18	0.03	1.12	to	1.24	.00
university A	1.15	0.24	0.71	to	1.85	.57
university B	1.14	0.39	0.53	to	2.45	.74
university C	1.09	0.31	0.60	to	1.98	.79
university D	0.65	0.52	0.24	to	1.79	.41
age	1.03	0.03	0.97	to	1.09	.31
sex (male vs. female)	0.95	0.18	0.67	to	1.35	.78

	HR	SE	95%CI			*P* value
CR[h]	1.18	0.18	0.83	to	1.68	.37
PHQ-9 Point	1.18	0.03	1.12	to	1.25	.00
university A	1.15	0.24	0.72	to	1.84	.57
university B	1.13	0.38	0.54	to	2.36	.75
university C	1.09	0.31	0.60	to	2.00	.78
university D	0.65	0.46	0.26	to	1.60	.34
age	1.03	0.03	0.97	to	1.09	.34
sex (male vs. female)	0.96	0.18	0.67	to	1.36	.80

	HR	SE	95%CI			*P* value
AT[i]	0.85	0.18	0.60	to	1.20	.35
PHQ-9 Point	1.18	0.03	1.12	to	1.25	.00
university A	1.14	0.24	0.72	to	1.82	.57
university B	1.13	0.38	0.54	to	2.38	.74
university C	1.08	0.31	0.59	to	1.98	.80
university D	0.65	0.48	0.26	to	1.66	.37
age	1.03	0.03	0.98	to	1.09	.29
sex (male vs. female)	0.96	0.18	0.68	to	1.35	.80

	HR	SE	95%CI			*P* value
PS[j]	1.18	0.18	0.82	to	1.68	.37
PHQ-9 Point	1.18	0.03	1.12	to	1.25	.00
university A	1.15	0.24	0.72	to	1.85	.55
university B	1.14	0.38	0.54	to	2.41	.74
university C	1.09	0.31	0.59	to	2.00	.79
university D	0.65	0.55	0.22	to	1.91	.43
age	1.03	0.03	0.97	to	1.09	.36
sex (male vs. female)	0.95	0.18	0.67	to	1.34	.77

[a]CBT: cognitive behavioral therapy.
[b]MDE: major depressive episode
[c]HR: hazard ratio.
[d]SE: standard error.
[e]SM: self-monitoring.
[f]PHQ-9: Patient Health Questionnaire-9.
[g]BA: behavioral activation.
[h]CR: cognitive restructuring.
[i]AT: assertiveness training.
[j]PS: problem-solving.
