# Supplementary material for: Long-Term Effects of Internet-Based Cognitive Behavioral Therapy on Depression Prevention Among University Students: Randomized Controlled Factorial Trial
Source: JMIR Ment Health. 2024 Sep 24;11:e56691. doi: 10.2196/56691 (PMC11445681; doi:10.2196/56691)
Supplement: Multimedia Appendix 4 [file mental-v11-e56691-s004.pdf]

Summary of the results of simple cox regression analyses of each CBT[a] component on the occurrence of MDE[b]

| Component | Cases with MDE, n/N (Completed N) | | HR[c] for incident MDE | | | | | |
|---|---|---|---|---|---|---|---|---|
| | Presence | Absence | HR | SE[d] | 95%CI | | | *P* value |
| SM[e] | 75 / 808 (638) | 58 / 818 (663) | 1.37 | 0.18 | 0.96 | to | 1.95 | .08 |
| BA[f] | 69 / 817 (648) | 64 / 809 (653) | 1.09 | 0.17 | 0.78 | to | 1.52 | .63 |
| CR[g] | 72 / 811 (652) | 61 / 815 (649) | 1.18 | 0.17 | 0.84 | to | 1.67 | .33 |
| AT[h] | 62 / 814 (647) | 71 / 812 (654) | 0.88 | 0.18 | 0.62 | to | 1.24 | .46 |
| PS[i] | 71 / 811 (646) | 62 / 815 (655) | 1.17 | 0.18 | 0.82 | to | 1.66 | .38 |

[a]CBT: cognitive behavioral therapy.
[b]MDE: major depressive episode
[c]HR: hazard ratio.
[d]SE: standard error.
[e]SM: self-monitoring.
[f]BA: behavioral activation.
[g]CR: cognitive restructuring.
[h]AT: assertiveness training.
[i]PS: problem-solving.
